# Association Between Macronutrients Intake and Depression in the United States and South Korea

**DOI:** 10.3389/fpsyt.2020.00207

**Published:** 2020-03-17

**Authors:** Jihoon Oh, Kyongsik Yun, Jeong-Ho Chae, Tae-Suk Kim

**Affiliations:** ^1^Department of Psychiatry, The Catholic University of Korea, Seoul, South Korea; ^2^Computation and Neural Systems, California Institute of Technology, Pasadena, CA, United States; ^3^Bio-Inspired Technologies and Systems, California Institute of Technology, Pasadena, CA, United States

**Keywords:** depression, macronutrients, nutritional psychiatry, National Health and Nutrition Examination Survey (NHANES), national survey

## Abstract

Although the risk for depression appears to be related to daily dietary habits, how the proportion of major macronutrients affects the occurrence of depression remains largely unknown. This study aims to estimate the association between macronutrients (i.e., carbohydrate, protein, fat) and depression through national survey datasets from the United States and South Korea. Association between the prevalence of depression and each macronutrient was measured from 60,935 participants from the National Health and Nutrition Examination Survey (NHANES) and 15,700 participants from the South Korea NHANES (K-NHANES) databases. When the proportion of calories intake by protein increased by 10%, the prevalence of depression was significantly reduced both in the United States [Odds Ratio, OR (95% CI), 0.621 (0.530–0.728)] and South Korea [0.703 (0.397–0.994)]. An association between carbohydrate intake and the prevalence of depression was seen in the United States [1.194 (1.116–1.277)], but not in South Korea. Fat intake was not significantly associated with depression in either country. Subsequent analysis showed that the low protein intake groups had significantly higher risk for depression than the normal protein intake groups in both the United States [1.648 (1.179–2.304)] and South Korea [3.169 (1.598–6.286)]. In the daily diet of macronutrients, the proportion of protein intake is significantly associated with the prevalence of depression. These associations were more prominent in adults with insufficient protein intake, and the pattern of association between macronutrients and depression in Asian American and South Korean populations were similar. Our findings suggest that the proportion of macronutrients intake in everyday life may be related to the occurrence of depression.

## Introduction

Dietary habits can affect physical health and illnesses. Changes in dietary patterns are related to the occurrence of many chronic diseases (1). A prudent dietary pattern (high consumption of vegetable, fruit, fish, poultry, and whole grains) was associated with a lower risk for type 2 diabetes (2), and low-risk dietary choices could prevent myocardial infarction (3). The adoption of Mediterranean diets (high consumption of fruits, vegetables, and mono-saturated fat) had a protective benefit against ischemic cerebral stroke (4), lowered the risk for cardiovascular disease (5), and had a beneficial effect in lowering the risk of death from all causes in a US population (6).

The importance of dietary patterns is also emphasized in the occurrence and prevention of psychiatric disorders (7–9). In Jacka et al. based on factor analysis, dietary patterns were categorized into three types (traditional, western, and modern), and they found that a traditional diet pattern, including the intake of vegetable, fruits, fish, and whole grains, was significantly associated with lower odds of depression, whereas a western diet pattern was related to a higher prevalence of depression (10). A prospective cohort study found that middle-aged adults with a “whole food” pattern, which is loaded with vegetables, fruits, and fish, had a lower risk of depression (11). Adherence to Mediterranean diets had a potential protective role on the occurrence of depressive disorders, and proinflammatory cytokines have been suggested as a possible mechanism for this association (12).

There are relatively few studies examining the relationship between depression and macronutrients, which are the basic constituents of daily diets, and the results remain inconsistent. One study that used the National Health and Nutrition Examination (NHANES) dataset, which was examined in 1971–1975, found that high protein intake lowers the risk for depression in adult men, but not in women (13). Another study also found that Japanese male workers who had low protein intake showed higher odds of depressive symptoms (14). Other studies also investigated the association between depression and macronutrient intake (15, 16) but failed to find significant correlations. These controversial results appear to be due to the lack of sample size and to selection bias (14).

Recent suggestions of dietary patterns have shifted toward adjusting the intake ratio of macronutrients (e.g., low-carbohydrate and high-protein diets) (17), and the effects on chronic illnesses and weight control have been reported (18, 19). However, how the proportions of macronutrient intake are related to the prevalence of depression in large populations is unknown. In the current study, we investigated the association of macronutrient intake ratios with depression in a general population. Nationwide nutrition and health survey datasets of the United States and South Korea were evaluated to compare the association between macronutrients and depression in two countries where the dietary habits and ethnic compositions are different.

## Materials and Methods

### Datasets and Covariates

The National Health and Nutrition Examination Survey datasets of the United States (NHANES, 2005 to 2016) and South Korea (K-NHANES, 2014 and 2016) were addressed in this analysis. The NHANES and K-NHANES are nationwide surveys that assess the health and nutritional status of the general population in each country. We recently analyzed the NHANES and K-NHANES datasets and classified depression (20). This two-year cross-sectional survey addresses complex, multistage, stratified sampling of the entire population. The NHANES consists of demographic, dietary, and other questionnaire data as well as a medical examination and various laboratory tests (21). The K-NHANES, which has been conducted since 1998, also has a complex, multistage stratification sample design and consists of similar categories as the NHANES (22).

Both the NHANES and K-NHANES have used the Patient Health Questionnaire 9 (PHQ-9), which consists of 9 self-administered questionnaires for screening depression. As the NHANES began using the PHQ-9 from the 2005–2006 survey, and the K-NHANES had a PHQ-9 item in 2014 and 2016 surveys, we targeted a 12-year-dataset of the NHANES (from 2005 to 2016) and a 2-year-dataset of the K-NHANES. A total of 60,935 (NHANES) and 15,700 (K-NHANES) individuals were enrolled to survey in these periods. As we used a pairwise method in the survey logistic regression procedure, the number of participants included in the analysis was related to both the dependent variable and covariates. In the NHANES, 15,296 of 60,935 participants had valid responses for all variables, and 7,942 of 15,700 participants satisfied these criteria (**Supplementary Figure 1**).

This study included the demographic covariates of age, sex, living with partner, and education level. Chronic illnesses such as hypertension, hypercholesterolemia, diabetes, history of chest pain, and history of cerebral stroke were also used as categorical covariates. Among these variables, education level and monthly income were modified to have a dichotomous value as these variables had different categories between the NHANES and K-NHANES [Education level: NHANES code (DMDEDU2), higher than a bachelor's degree or not; K-NHANES code (edu), higher than a bachelor's degree or not. Monthly income: NHANES code (IND235), over $ 2,900 or not; K-NHANES code (incm), above 25^th^ percentile or not]. Since the independent variable of three macronutrients (ratio of carbohydrate, protein, and fat intake) are collinear, only one variable was used in the survey logistic regression analysis.

### Outcome Measures

The measurement of depression in the NHANES and K-NHANES datasets was identical. Both datasets used the PHQ-9 for screening for depression and a cut-off value of 10 was the threshold for the diagnosis of depression, which reflects moderate to severe depression (23). The PHQ-9 is mainly based on the diagnostic criteria of depression from the Diagnostic and Statistical Manual of Mental Disorders IV (DSM-IV) (24), and it is known to be a reliable and valid measurement in screening for depression in general population (25).

### Dietary Requirement for Macronutrients

The recommended dietary intake of macronutrients varies across countries. In the United States, we followed the Acceptable Macronutrient Distribution Range (AMDR) guideline (26). The AMDR recommends intakes of macronutrients as percentages of the total daily energy intake. The AMDR suggests carbohydrate intake be between 45 to 60 percent of the daily energy intake and protein intake 10 to 35 percent. Recommended fat intake ranges from 20 to 35%.

In South Korea, the Dietary Reference Intakes for Koreans (KDRIs) guideline suggests the intake of carbohydrates as should be between 55 to 70 percent of the total daily energy intake (27). The range of suggested protein intake is 10 to 20 percent and those of fat intake is 15 to 25 percent. According to these guidelines, we classified low-normal-high protein intake groups and normal carbohydrate intake group in the NHANES and K-NHANES datasets.

### Statistical Analysis

The complex samples survey logistic regression method was used in further analyses. Multiple stratification variables of each dataset were addressed in the analysis, and missing values were treated as valid (28). The missing rate of included variables ranged from 0 to 5.6 percent. In NHANES, the data were weighted with the exam weight (wtmec2yr), which represents the weight of each two-year survey dataset. In K-NHANES, two stages of stratified clustering were applied, consisting of primary sampling units and households. The weights of “wt_itvex” were applied to the analysis, and the weights for 2014 and 2016 were reduced by half and summed (29, 30). Domain analysis was used to calculate estimates for sub-population in both the NHANES and K-NHANES datasets. Data collection and trimming were performed using SPSS version 18.0 (SPSS Inc., Chicago, IL), and survey logistic procedures were performed with SAS statistical software version 9.4 (SAS Institute, Inc. Cary, NC).

### Linear Regression and Subgroup Analysis

To visualize the correlation between dietary intake and depression, we scatter-plotted raw data of the PHQ-9 total score and each macronutrient intake ratio (**Supplementary Figure 2**). In this graph, the radius of each data-point represents its weight value, and linear regression was performed to confirm the general association between PHQ-9 total score and macronutrient intake ratio. Therefore, the slope and the y-axis intercept values of linear regression were not adjusted for covariates.

**Figure 1:**
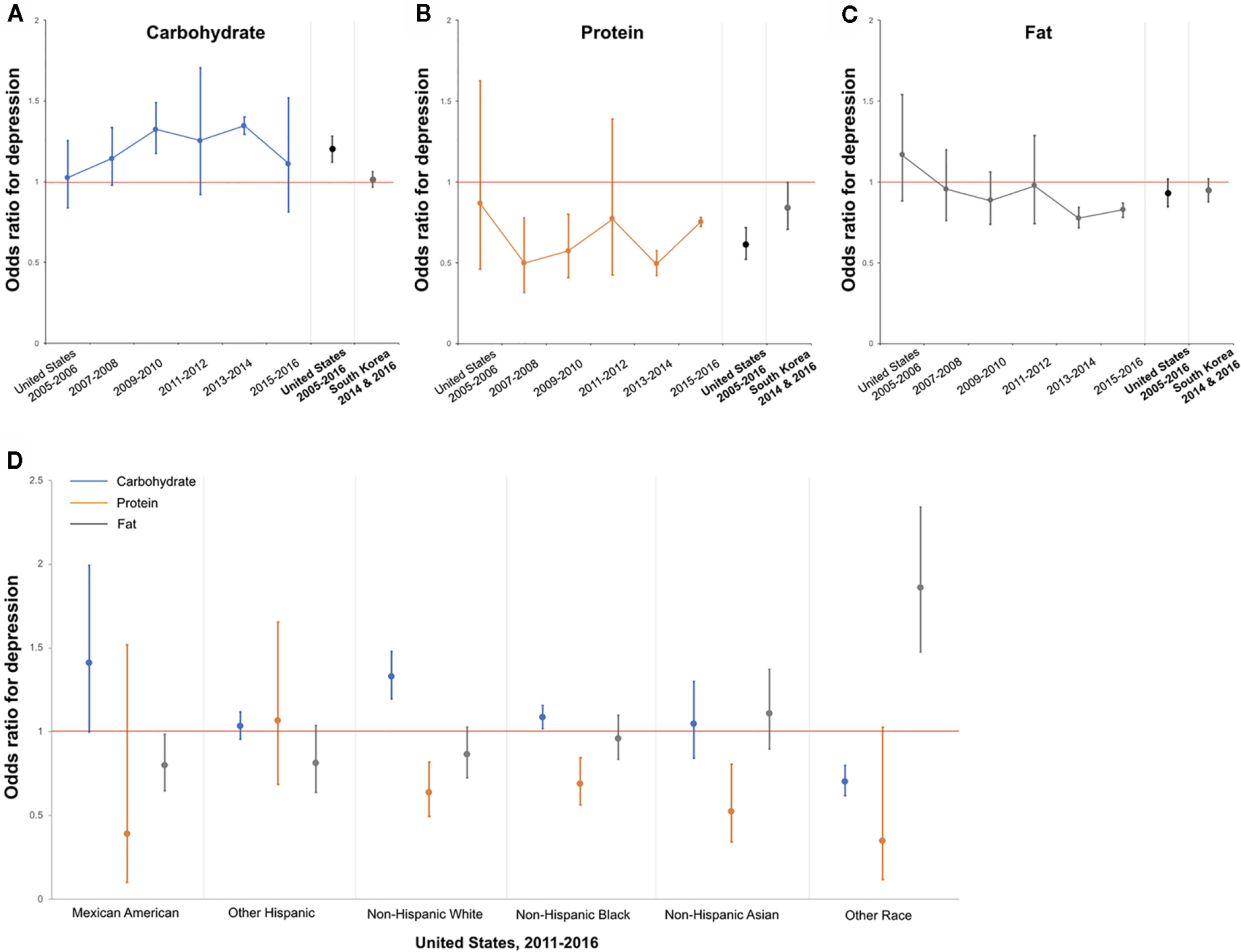
Association between Protein Intake and Depression in the United States and South Korea—Trends based on 2-Year Datasets and Race Groups. **(A)** Odds ratio for depression as calories from carbohydrate increase by 10 percent. The results from 2-year datasets and pooled results of the United States and South Korea. **(B)** Protein. **(C)** Fat. **(D)** Odds ratio for depression as each macronutrient increase by 10 percent in each race/ethnic group. The results based on the pooled 2011–2016 NHANES datasets. Red line denotes an odds ratio of one. Error bars represent 95% CI.

To confirm the effect of race on the observed associations, we performed survey logistic regression with the race/ethnicity (NHANES dataset code: RIDRETH3) as a domain variable. As the K-NHANES only targeted the Asian population, we aimed to compare the results of the NHANES and the K-NHANES datasets. The non-Hispanic Asian category was newly added in the 2011–2012 NHANES survey, and this domain analysis included 2011 to 2016 survey data of the NHANES (**Figure 1D**). Covariates and dependent variables were identical for all year analyses.

## Results

### Sample Characteristics

In the United States, a total of 60,935 participants were recruited for analysis, and 15,296 individuals who were evaluated for depression were eligible for further analysis. The prevalence rate for depression in this group was 7.7%. In South Korea, 15,700 participants were enrolled, and 7,942 were assessed for depression. The prevalence rate for depression was 6.3% when using the same criteria and cut-off score of 10 as for the United States (**Supplementary Figure 1**).

The sample characteristics of these datasets are presented in **Table 1**. All categorical variables, including a history of chronic illnesses, monthly income, living with partner, and education level were significantly different between the depression and non-depression groups. In the United States, the prevalence of hypertension, hypercholesterolemia, diabetes, history of chest pain, and cerebral stroke were higher in the depression group than in the non-depression groups. Participants with depression had a lower monthly income and lower level of education and higher rates of living alone than the non-depression group (**Table 1**). Similar findings were found in the dataset of South Korea except for hypercholesterolemia; the depression group showed a slightly lower prevalence of hypercholesterolemia than the non-depression group [depression vs. non-depression, mean (95% CI); 13.2% (7.8–21.6) vs. 14.6% (13.2–16.1)].

**Table 1:** Selected characteristics of the weighted sample.

United States, NHANES 2005-2016				
	Men (*n* = 7,472; *n_w_* = 5.05 x 10^7^)		Women (*n* = 7,824; *n_w_* = 5.63 x 10^7^)	
	No depression (*n* = 7,053; *n_w_* = 4.81 x 10^7^)	Depression (*n* = 419; *n_w_* = 2.39 x 10^6^)	No depression (*n* = 7,070; *n_w_* = 5.19 x 10^7^)	Depression (*n* = 754; *n_w_* = 4.45 x 10^6^)
**Categorical variables** **Percent, (95% CI)**				
High blood pressure, yes	43.7 (41.9-45.6)	54.3 (47.1-61.2)	44.2 (42.5-45.9)	58.4 (53.7-63.0)
High cholesterol level, yes	49.6 (48.0-51.3)	53.6 (46.3-60.7)	45.0 (43.5-46.5)	55.3 (50.7-59.8)
Diabetes, yes	14.7 (13.5-15.9)	18.7 (15.0-23.2)	11.8 (10.9-12.9)	23.5 (19.9-27.6)
History of chest pain, yes	26.1 (24.7-27.6)	52.0 (44.5-59.5)	24.2 (23.0-25.5)	48.6 (43.9-53.4)
Cerebral stroke, yes	3.7 (3.2-4.4)	9.6 (6.5-14.1)	3.7 (3.2-4.3)	8.8 (6.4-11.9)
Monthly income (≥ $2,900)	68.6 (61.1-76.7)	40.2 (22.1-72.1)	59.5 (57.3-61.5)	38.0 (33.2-42.9)
Living with partner, yes	76.9 (75.1-78.6)	54.4 (47.8-60.9)	63.5 (44.5-62.0)	44.5 (40.3-48.8)
Education level, bachelor's degree	63.4 (60.9-65.8)	41.1 (34.7-47.9)	63.6 (61.3-65.7)	48.7 (43.6-53.8)
**Continuous variables** **Mean, (95% CI)**				
Age (year)	57.4 (57.0-57.8)	56.2 (55.0-57.4)	58.3 (57.9-58.7)	55.8 (54.7-56.8)
Dietary energy intake (kcal/day)	2429.7 (2396.3-2463.1)	2342.4 (2201.9-2482.9)	1766.6 (1744.7-1788.4)	1698.3 (1634.1-1762.5)
Energy intake from carbohydrates (%)	47.7 (47.4-48.1)	48.7 (47.0-50.3)	48.5 (48.1-49.0)	51.6 (50.3-52.8)
Energy intake from proteins (%)	15.9 (15.7-16.0)	14.8 (14.3-15.4)^†^	15.9 (15.7-16.0)	14.5 (14.0-15.1)^†^
Energy intake from fats (%)	34.6 (34.3-34.9)	33.7 (32.5-34.9)	34.6 (34.3-35.0)	33.7 (32.7-34.8)
BMI (kg/m^2^)	29.4 (29.2-29.6)	29.5 (28.7-30.4)	29.4 (29.2-29.7)	32.9 (32.1-33.6)^†^
PHQ-9 total score	1.7 (1.6-1.8)	13.5 (13.1-13.9)^†^	2.3 (2.2-2.4)	13.6 (13.3-13.9)^†^

**South Korea, K-NHANES 2014 and 2016**				
	**Men** **(*n* = 3,090; *n_w_* = 1.07 x 10^7^)**		**Women** **(*n* = 4,852; *n_w_* = 1.39 x 10^7^)**	
	**No depression** **(*n* = 2,975;** ***n_w_* = 1.04 x 10^7^)**	**Depression** **(*n* = 115;** ***n_w_* = 3.66 x 10^5^)**	**No depression** **(*n* = 4,469;** ***n_w_* = 1.28x 10^7^)**	**Depression** **(*n* = 383;** ***n_w_* = 1.06 x 10^6^)**
**Categorical variables** **Percent, (95% CI)**				
High blood pressure, yes	24.5 (22.7-26.3)	36.4 (26.4-47.7)	20.3 (18.9-21.8)	31.3 (26.3-36.7)
High cholesterol level, yes	14.6 (13.2-16.1)	13.2 (7.8-21.6)	15.7 (14.5-17.0)	24.7 (20.0-30.0)
Diabetes, yes	10.0 (8.9-11.2)	23.1 (14.8-34.2)	7.2 (6.4-8.2)	15.9 (12.1-20.7)
History of chest pain, yes	3.0 (2.5-3.6)	5.9 (2.9-11.8)	1.7 (1.4-2.2)	7.8 (5.2-11.5)
Cerebral stroke, yes	2.2 (1.7-2.8)	9.8 (5.1-18.1)	1.3 (1.0-1.7)	4.9 (3.2-7.5)
Monthly income (> 25% percentile)	27.0 (24.6-29.5)	16.6 (10.1-26.0)	27.0 (12.4-25.8)	12.4 (8.9-17.2)
Living with partner, yes	93.8 (92.6-94.8)	78.7 (69.0-85.9)	83.6 (82.1-84.9)	61.9 (56.4-67.1)
Education level, bachelor's degree	48.3 (45.5-51.1)	35.0 (23.8-48.0)	35.1 (33.0-37.2)	20.7 (16.2-26.0)
**Continuous variables** **Mean, (95% CI)**				
Age (year)	52.4 (51.7-53.0)	52.7 (49.3-56.1)	51.3 (50.7-52.0)	55.3 (53.3-57.3)
Dietary energy intake (kcal/day)	2395.7 (2353.9-2437.5)	2388.4 (2157.7-2619.1)	1714.7 (1689.2-1740.1)	1545.2 (1462.4-1628.0)
Energy intake from carbohydrates (%)	60.6 (60.0-61.2)	59.0 (55.9-62.1)	65.5 (65.0-66.0)	68.5 (67.0-70.1)
Energy intake from proteins (%)	13.9 (13.8-14.1)	13.0 (11.7-14.3)	13.9 (13.8-14.1)	13.2 (12.8-13.6)
Energy intake from fats (%)	18.5 (18.2-18.9)	17.0 (15.4-18.7)	18.8 (18.5-19.2)	16.7 (15.6-17.8)
BMI (kg/m^2^)	24.5 (24.4-24.6)	23.7 (23.0-24.4)	23.6 (23.4-23.7)	24.0 (23.6-24.5)
PHQ-9 total score	1.5 (1.5-1.6)	13.5 (12.7-14.2)^†^	2.2 (2.2-2.3)	14.1 (13.6-14.5)^†^

BMI, body mass index; PHQ-9, Patient Health Questionnaire; CI, Confidence Interval; n, unweighted number of samples; n_w_, weighted number of samples.

^†^p < 0.001.

All categorical variables were significantly different between depression and non-depression.

In both the United States and South Korea, age and daily energy intake were not significantly different between the depression and non-depression groups (**Table 1**). BMI was significantly higher in women with depression in the United States [depression vs. non-depression, mean (95% CI); 32.9 (32.1–33.6) vs. 29.4 (29.2–29.7)], but there was no significant difference in South Korean women.

The proportion of daily calorie intake from each macronutrient also varied largely between the two countries (**Supplementary Figure 3**). In the United States, 33.4% of daily calories were derived from protein, but only 13.9% of calories were taken from protein in South Korea. Carbohydrates accounted for 50.2% of calories consumed per day in the United States and for 61.3% in South Korea. In both datasets, carbohydrates were the primary source of energy, but the proportions of each macronutrient varied widely.

### Adjusted Association Between Each Macronutrient and Depression

**Tables 2** and **3** present adjusted associations between each macronutrient and depression in the United States and South Korea, respectively. The odds ratio (OR) for depression was associated with increased protein intake in all adults of both countries. As the calorie intake from protein increased by 10%, the prevalence of depression was significantly decreased in the United States (OR, 0.621; 95% CI, 0.530–0.728, **Table 2**) and in South Korea (OR, 0.703; 95% CI, 0.497–0.994, **Table 3**). These findings were significant when dividing the dataset by gender in the United States (Men; OR, 0.681; 95% CI, 0.550–0.843, Women; OR, 0.597; 95% CI, 0.472–0.754), but were not significant in South Korea.

**Table 2:** Adjusted association between calories from each nutrient and depression in United States, 2005-2016.

	Carbohydrates (%)^†^	Proteins (%)	Fats (%)
**Depression Status**			
**All adults**			
No Depression	47.7 (47.4-48.0)	15.9 (15.8-16.0)	34.4 (34.2-34.6)
Depression	50.9 (50.0-51.7)	14.4 (14.0-14.7)	33.2 (32.5-33.8)
Odds Ratio (95% CI)^‡^	1.194 (1.116-1.277)^¶^	0.621 (0.530-0.728)^¶^	0.923 (0.840-1.014)
**Men**			
No Depression	46.7 (46.3-47.0)	16.0 (15.8-16.2)	34.3 (34.0-34.5)
Depression	49.3 (47.6-51.0)	14.6 (14.0-15.1)	32.8 (31.7-33.9)
Odds Ratio (95% CI)^§^	1.144 (0.993-1.317)	0.681 (0.550-0.843)^¶^	0.976 (0.863-1.103)
**Women**			
No Depression	48.7 (48.4-49.1)	15.7 (15.6-15.9)	34.5 (34.3-34.8)
Depression	51.7 (50.6-52.7)	14.3 (13.9-14.7)	33.4 (32.5-34.2)
Odds Ratio (95% CI)^§^	1.223 (1.127-1.328)^¶^	0.597 (0.472-0.754)^¶^	0.895 (0.799-1.002)

^†^(Calories from each nutrient)/(total calories intake) x 100 (%).

^‡^Adjusted for age, gender, marital status, education, income, BMI, hypertension, dyslipidemia, diabetes and chest pain.

^§^Adjusted for age, marital status, education, income, BMI, hypertension, dyslipidemia, diabetes and chest pain.

^¶^p < 0.001.

**Table 3:** Adjusted association between calories from each nutrient and depression in South Korea, 2014 and 2016.

Depression Status	Carbohydrates (%)^†^	Proteins (%)	Fats (%)
**All adults**			
No Depression	63.3 (62.9-63.7)	13.9 (13.8-14.1)	18.7 (18.4-19.0)
Depression	66.1 (64.7-67.5)	13.1 (12.7-13.6)	16.8 (15.8-17.7)
Odds Ratio (95% CI)^‡^	1.019 (0.929-1.118)	0.703 (0.497-0.994)^¶^	0.894 (0.769-1.039)
**Men**			
No Depression	60.6 (60.0-61.2)	13.9 (13.8-14.1)	18.5 (18.2-18.9)
Depression	59.0 (55.9-62.1)	13.0 (11.7-14.3)	17.0 (15.4-18.7)
Odds Ratio (95% CI)^§^	0.878 (0.765-1.008)	0.705 (0.274-1.809)	0.925 (0.683-1.252)
**Women**			
No Depression	65.5 (65.0-66.0)	13.9 (13.8-14.1)	18.8 (18.5-19.2)
Depression	68.5 (67.0-70.1)	13.2 (12.8-13.6)	16.7 (15.6-17.8)
Odds Ratio (95% CI)^§^	1.111 (0.983-1.256)	0.732 (0.524-1.021)	0.895 (0.758-1.056)

^†^(Calories from each nutrient)/(total calories intake) x 100 (%).

^‡^Adjusted for age, sex, marital status, income, BMI, hypertension, dyslipidemia, diabetes and chest pain.

^§^Adjusted for age, marital status, income, BMI, hypertension, dyslipidemia, diabetes and chest pain.

^¶^p < 0.05.

Among the other macronutrients, carbohydrates showed a significant association with depression in the United States. As the calories from carbohydrates increased by 10%, the prevalence of depression increased (OR, 1.194; 95% CI, 1.116–1.277). However, there was no significant association between carbohydrates and depression in South Korea's dataset. Fat did not show significant associations in either country (**Tables 2** and **3**).

Linear regression analysis of three macronutrients and the PHQ-9 total score also showed these associations (**Supplementary Figure 3**). Although these results were not adjusted for covariates, a negative linear correlation between the ratio of protein intake and PHQ-9 total score was seen with statistical significance (p < 0.05) in both countries. Carbohydrates had a positive correlation with the PHQ-9 total score in both countries' datasets but did not show statistical significance.

### Adjusted Association Between Protein Intake Status and Depression

To assess whether these associations were related to sufficient or lack of protein intake, we divided the participants into three groups according to their protein intake status. As the dietary recommendation for each macronutrient varied between the United States and South Korea, we followed the dietary guideline in each country (see *Methods* section for details). The results of adjusted associations between protein intake status and depression are presented in **Table 4**.

**Table 4:** Adjusted association between protein groups and depression in United States and South Korea.

Protein Intake Status	All adults^¶^	Men^§^	Women^§^
**United States**^†^			
Low protein intake	1.648 (1.179-2.304)^f^	0.950 (0.717-1.260)	2.025 (1.313-3.124)^***^
Normal	1 [reference]	1 [reference]	1 [reference]
High protein intake	0.801 (0.223-2.879)	0.212 (0.030-1.523)	1.300 (0.353-4.785)
**South Korea**^‡^			
Low protein intake	3.169 (1.598-6.286)^**^	4.924 (1.411-17.182)^*^	2.347 (0.951-5.920)
Normal	1 [reference]	1 [reference]	1 [reference]
High protein intake	0.896 (0.581-1.383)	0.695 (0.254-1.897)	0.962 (0.597-1.552)

^†^Depending on the ratio of calories from protein to total energy intake (<10%:low protein intake, 10~34%:normal, >35%: high protein intake).

^‡^Depending on the ratio of calories from protein to total energy intake (<7%:low protein intake, 7~20%:normal, >20%: high protein intake).

^§^Adjusted for age, gender, marital status, income, BMI, hypertension, dyslipidemia, diabetes and chest pain.

^¶^Adjusted for age, marital status, income, BMI, hypertension, dyslipidemia, diabetes and chest pain.

***p < 0.001; **p < 0.01; *p < 0.05.

In both countries, the low protein intake groups had higher odds for depression than the normal protein intake groups; (United States, OR, 1.658; 95% CI, 1.179–2.304, South Korea, OR, 3.169; 95% CI, 1.598–6.286). However, the high protein intake groups did not show significant associations with the prevalence of depression in either dataset. These results suggest that observed associations between protein intake and depression might be related to a lack of protein intake rather than an excess of protein intake.

In the United States dataset, carbohydrates were also found to be associated with depression. Therefore, we further analyzed how protein intake is related to depression in participants who ingest carbohydrates in the normal range (**Supplementary Table 1**). The analysis showed that the prevalence of depression significantly decreased as the calories from protein increased by 10% in the normal carbohydrate intake group (OR, 0.603; 95% CI, 0.498–0.729). In South Korea, similar findings were found, although the results failed to reach statistical significance (OR, 0.569, 95% CI, 0.310–1.042, p-value = 0.067). Fat did not show any significant associations in either countries' datasets. These findings suggest that the association between protein intake and depression might not be due to the change in the composition ratio of other macronutrients, as the protein increases or decreases.

### Trend by 2-Years and Race Groups

**Figure 1** represents the trend of the association between protein intake and depression by 2-years datasets and race groups. Among 6 sub-datasets paired for 2 years, only protein had a relatively consistent association with depression: 2007–2010 and 2013–2016 (4 of 6 sub-datasets, **Figure 1B**). Although carbohydrate intake showed a significant association with depression in the pooled dataset of all years (2005–2016), there was no significant correlation in most of the years (significant in 2 of 6 sub-datasets, **Figure 1A**).

To investigate whether race and ethnicity affect the observed associations, we performed domain analysis by race/ethnicity in the United States' dataset. The analysis showed that the association between protein intake and depression varied by race and ethnicity. Mexican American and Other Hispanic individuals did not show significant associations, but Non-Hispanic White, Non-Hispanic Black, and Non-Hispanic Asian individuals had significant associations between increased protein intake and a decreased prevalence of depression. Non-Hispanic Asian individuals in the United States had a similar pattern of association with South Korea, which is a single racial group; among the three macronutrients, only protein showed a significant association with depression (**Figure 1D**).

## Discussion

Our study suggests that the proportion of protein intake in the daily diet is highly associated with the prevalence of depression. Of the three macronutrients, only protein showed a significant correlation with depression in both countries. In the United States, this association was preserved when the subjects were limited to those with normal carbohydrate intake. In South Korea, statistically nonsignificant results for the normal carbohydrate intake group were found, but the similar odds ratio to the United States' dataset and near-threshold p-value might warrant further examination (**Supplementary Table 1**). The association between protein intake and depression varied between racial groups, and similar patterns of protein-depression relationship between non-Hispanic Asians in the United States and South Koreans in Korea were observed.

Similar findings were observed in the National Health and Nutrition Evaluation Survey Epidemiologic Follow-up Study (NHEFS) dataset (13). Wolfe et al., followed-up 10 years data of a NHEFS cohort from 1970s to 1980s and found that the highest third protein intake group was more likely to have less depression than the lowest third group (Relative Risk, 0.38; 95% CI, 0.16–0.92). As the NHEFS cohort was based on the participants of the NHANES, this finding suggests that the association between protein and depression might not be an epochal event but might have continuing relevance. Although we did not find a significant association in the 2005–2006 and 2011–2012 NHANES datasets, other datasets and pooled analysis revealed that increased protein intake was associated with a low prevalence of depression (**Figure 1**).

Previous studies that observed the association between macronutrients and depression showed somewhat conflicting results. Some studies found significant correlation (13, 14), but others did not (12, 15, 31). However, this discrepancy might be related to the small number of participants (n = 100 to 150) or limited numbers of participants of certain groups (e.g., elderly). As both the NHANES and K-NHANES targeted each countries' entire population, the observed associations in our study suggest that the lack of protein intake in the daily diet might be associated with a high prevalence of depression.

Similar results between participants in the United States and South Korea are noteworthy, as the daily dietary composition of these two countries were quite different. The proportion of calories from protein in the daily diet was more than two times higher in the United States than in South Korea (33.4 vs. 13.9%, **Supplementary Figure 3**). This difference is related to the traditional Korean dietary pattern characterized by consumption of rice and kimchi (32). As the total calorie intake was not largely different between the two countries (**Table 1**), the calorie amount from protein was quite higher in the United States than in Korea. Nevertheless, an increase of protein intake by 10% significantly lowered the risk for depression in both countries. Therefore, the relative composition of macronutrients, rather than the absolute intake of calories, might be associated with the development and prevention of depression.

Subsequent analyses showed that this association is related to race/ethnicity. Non-Hispanic Asians in the United States had a similar pattern to South Koreans, showing a significant association between protein intake and depression (**Figure 1D**). As mentioned above, these two groups differ in dietary patterns, macronutrients intake, and cultural backgrounds except that they are of the same race. Therefore, the correlation of depression with increasing proportion of protein intake could be related to genetic background, rather than environmental factors, such as dietary patterns and absolute intake of certain nutrients.

Several molecular hypotheses have been suggested regarding protein intake and depression. These hypotheses are based on the fact that amino acid, a constitute of protein, is related to tryptophan, which is a precursor of serotonin. Although the high protein intake could increase the plasma concentration of tryptophan (33), other neural amino acids can compete with tryptophan for uptake into brain (34). Therefore, increasing protein intake does not necessarily increase the tryptophan level in the brain. Due to this conflicting effect of protein intake on the tryptophan concentration, there is a difficulty in interpreting the result that an increased intake of protein prevents depression by increasing serotonin in the brain. Furthermore, other macronutrients can regulate the concentration and synthesis of tryptophan; the ingestion of carbohydrates or the injection of insulin is known to increase the plasma tryptophan concentrations (35). Given that several macronutrients are involved in the regulation of the tryptophan concentration, understanding the molecular mechanism of the association between protein intake and depression might need controlled experiments. Our findings might suggest implications for these future studies of the dietary effects on depression.

### Strengths and Limitations

This study has several strengths. First, the study included a large sample size and used nationwide survey datasets which targeted entire population. Therefore, the observed association between depression and protein intake could be applied to the general population. Second, we used datasets with the most accurate information on nutrition. Both the NHANES and K-NHANES datasets include over 400 items in the dietary questionnaire, and the amount of each macronutrient is calculated by these items. Third, comparison of the NHANES and K-NHANES datasets enabled us to compare the effect of race/ethnicity on the association, with remaining cultural factors and dietary patterns as confounding factors.

This study has several limitations, and caution should be used when interpreting the results. First, because we only measured the binary status of depression, it was difficult to evaluate how the severity of depression is affected by the degree of macronutrient intake. Second, PHQ-9 has been validated for detecting major and sub-threshold depression in the general population (36), but it is not a depression diagnostic tool. Third, no association between depression and micronutrients such as EPA (Eicosapentaenoic acid) and omega-3 was observed, which appears to be related to depression (37). In addition, the study did not evaluate certain types of macronutrients (starch, sugar, or carbohydrate fibers). Since this information was not used as a covariate, the present results did not rule out the possibility that reported association was confounded by micronutrient intake status. Fourth, the intake of each macronutrient is based on the total diet intake the day before the survey. This 24-h recall method is widely used in nutrition research (38), but could bias the estimation of usual dietary intake. Finally, since both datasets are cross-sectional surveys, there are no subsequent datasets. Therefore, we could not reveal the casual link between macronutrients and depression. To explain the causal effects of macronutrients on the occurrence of depression, we need to investigate the gradual changes in macronutrients intake that affect the probability of depression. Further research on cohort datasets can confirm that this association persists.

## Conclusions

Our study suggests that increasing calories from protein in the daily diet is associated with a lower prevalence of depression in adults in the United States and South Korea. When considering the dietary recommendations in each country, lower protein intake than the recommended amount significantly associated with the higher prevalence of depression. This association was similarly observed in the same ethnic groups in the two countries where dietary patterns and the proportion of nutrient intake were quite different. Our findings suggest that the proportion of daily intake of macronutrients may be related to the occurrence of depression. Further research regarding the longitudinal association between protein intake and the occurrence of depression in the other countries' datasets is needed.

## Data Availability Statement

The datasets generated for this study are available on request to the corresponding author.

## Ethics Statement

The studies involving human participants were reviewed and approved by the Institutional Review Board of the Ethics Committee of Seoul St. Mary's Hospital at The Catholic University of Korea (KC17ZESI0132). The patients/participants provided their written informed consent to participate in this study.

## Author Contributions

JO and T-SK conceived the idea of this study. JO and KY collected the data and performed the statistical analysis. JO drafted the manuscript. J-HC and T-SK reviewed the data and revised the manuscript.

## Funding

This research was supported by a grant of the Korea Health Technology R&D Project through the Korea Health Industry Development Institute (KHIDI), funded by the Ministry of Health & Welfare, Republic of Korea (grant number: HM15C1054).

## Conflict of Interest

The authors declare that the research was conducted in the absence of any commercial or financial relationships that could be construed as a potential conflict of interest.

## Supplementary Material

Supplementary Figure 1 Participant selection in NHANES and K-NHANES data sets.

Supplementary Figure 2 Proportion of macronutrients intake in daily dietary **(A)** United States **(B)** South Korea.

Supplementary Figure 3 Weighted scatter plots of PHQ-9 total score and each macronutrients intake ratio.
