# Supplementary figures and images for: Association Between Macronutrients Intake and Depression in the United States and South Korea
Source: Front Psychiatry. 2020 Mar 17;11:207. doi: 10.3389/fpsyt.2020.00207 (PMC7090018; doi:10.3389/fpsyt.2020.00207)

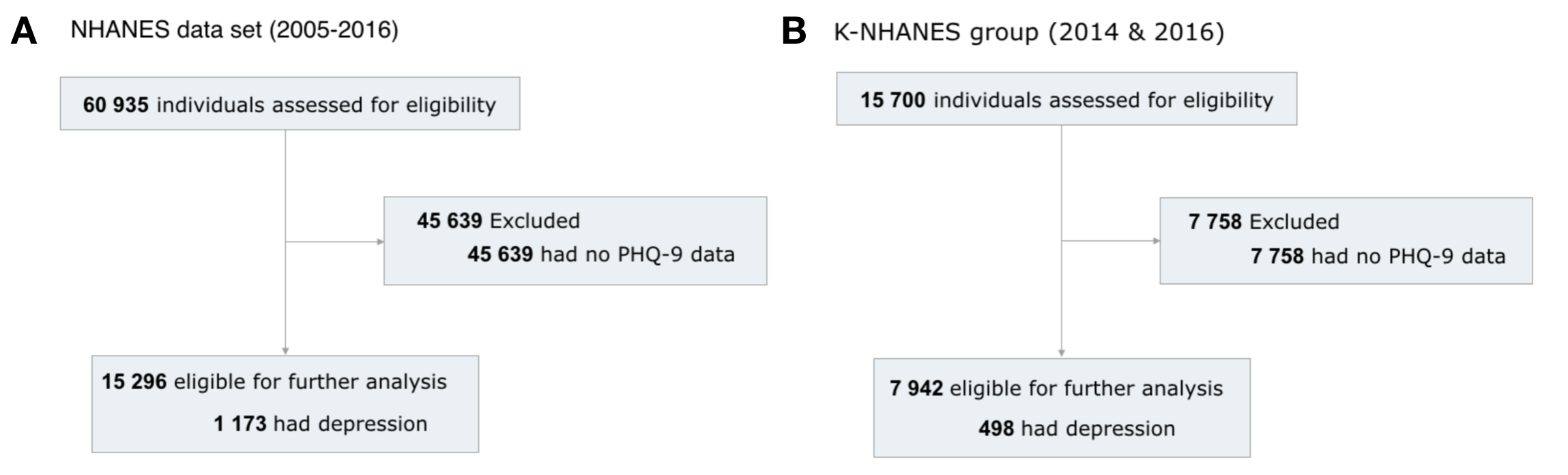

Supplement: Supplementary Figure 1 — Participant selection in NHANES and K-NHANES data sets. [file Image_1.png]

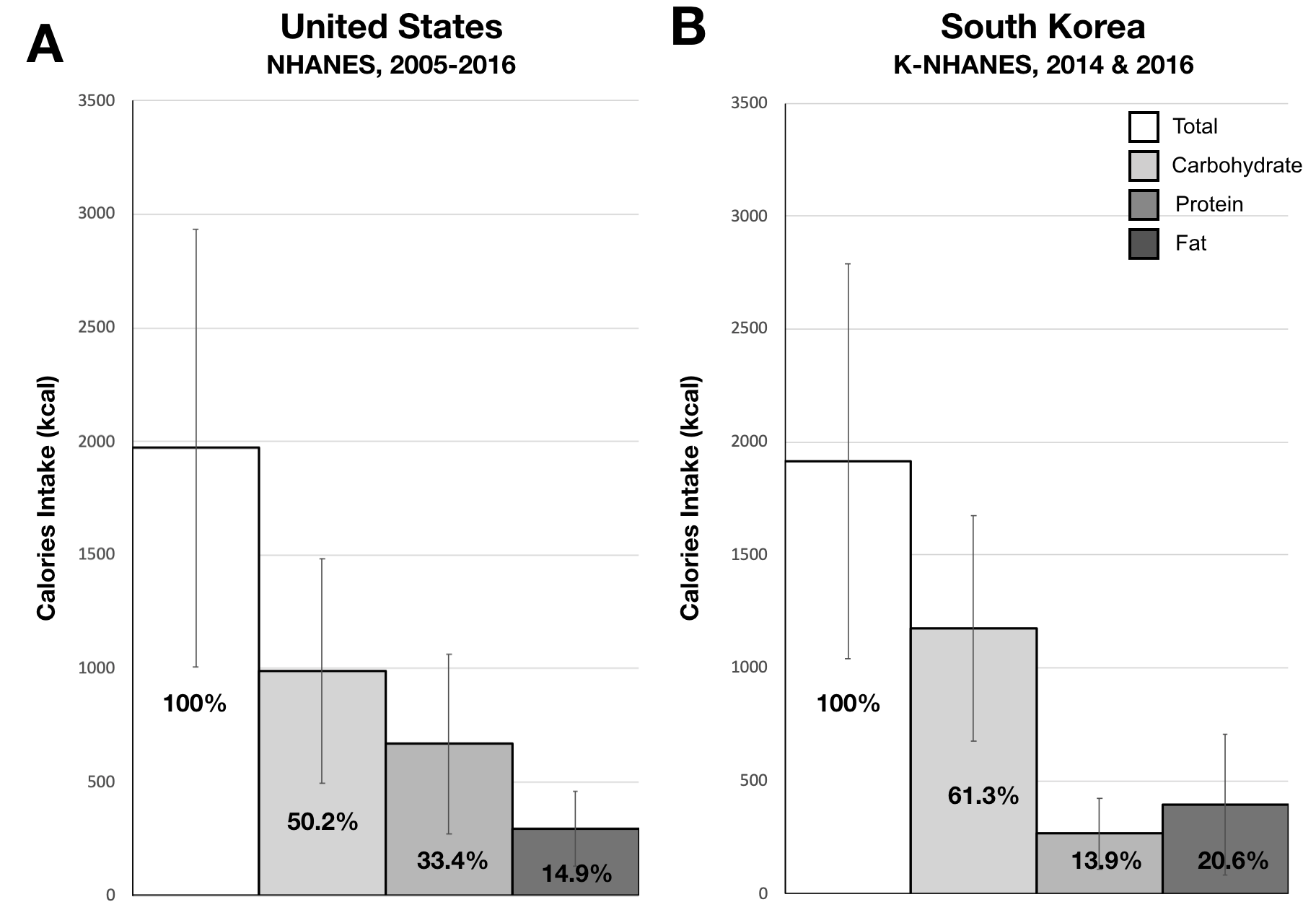

Supplement: Supplementary Figure 2 — Proportion of macronutrients intake in daily dietary (A) United States (B) South Korea. [file Image_2.png]

Carbs

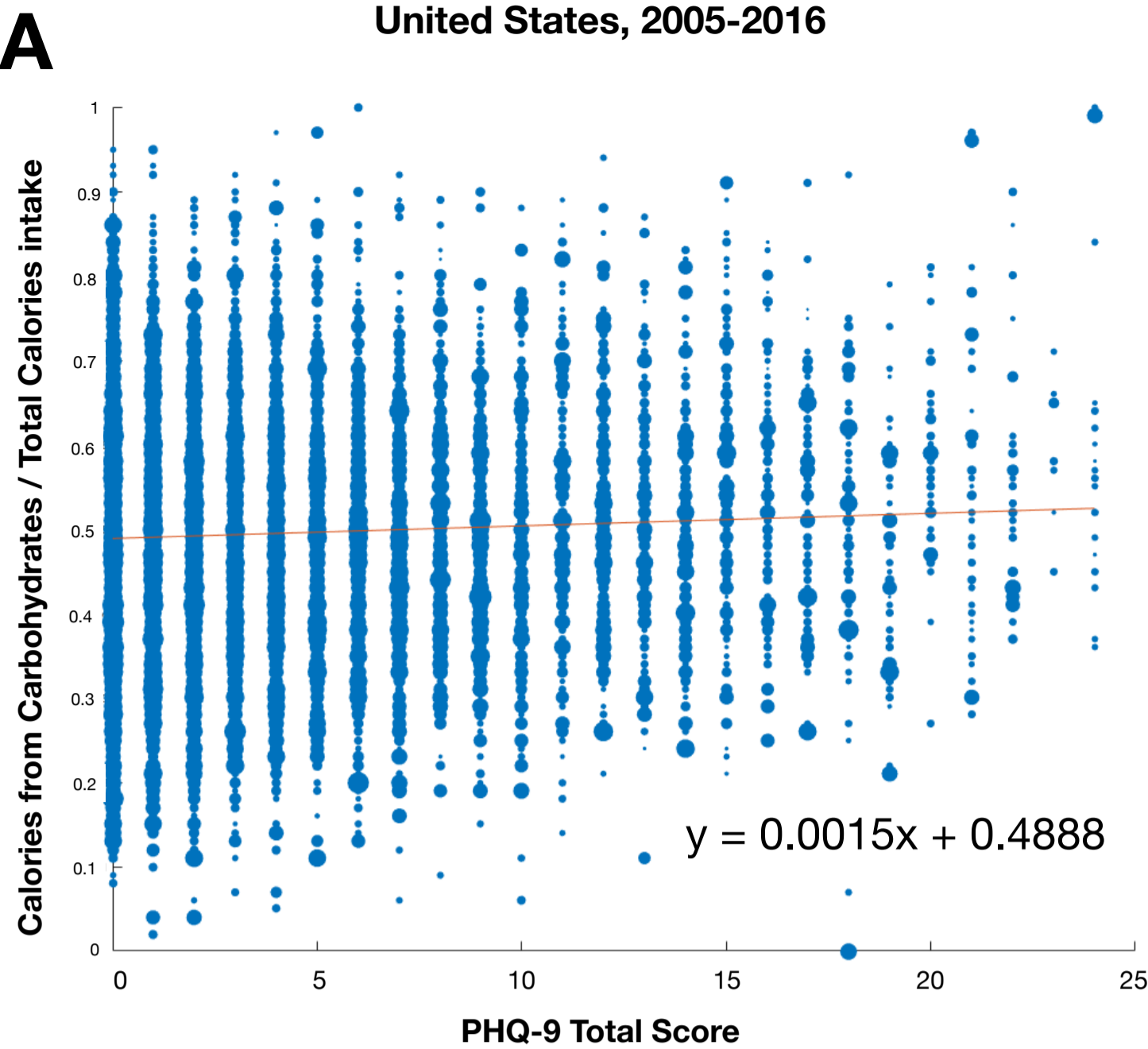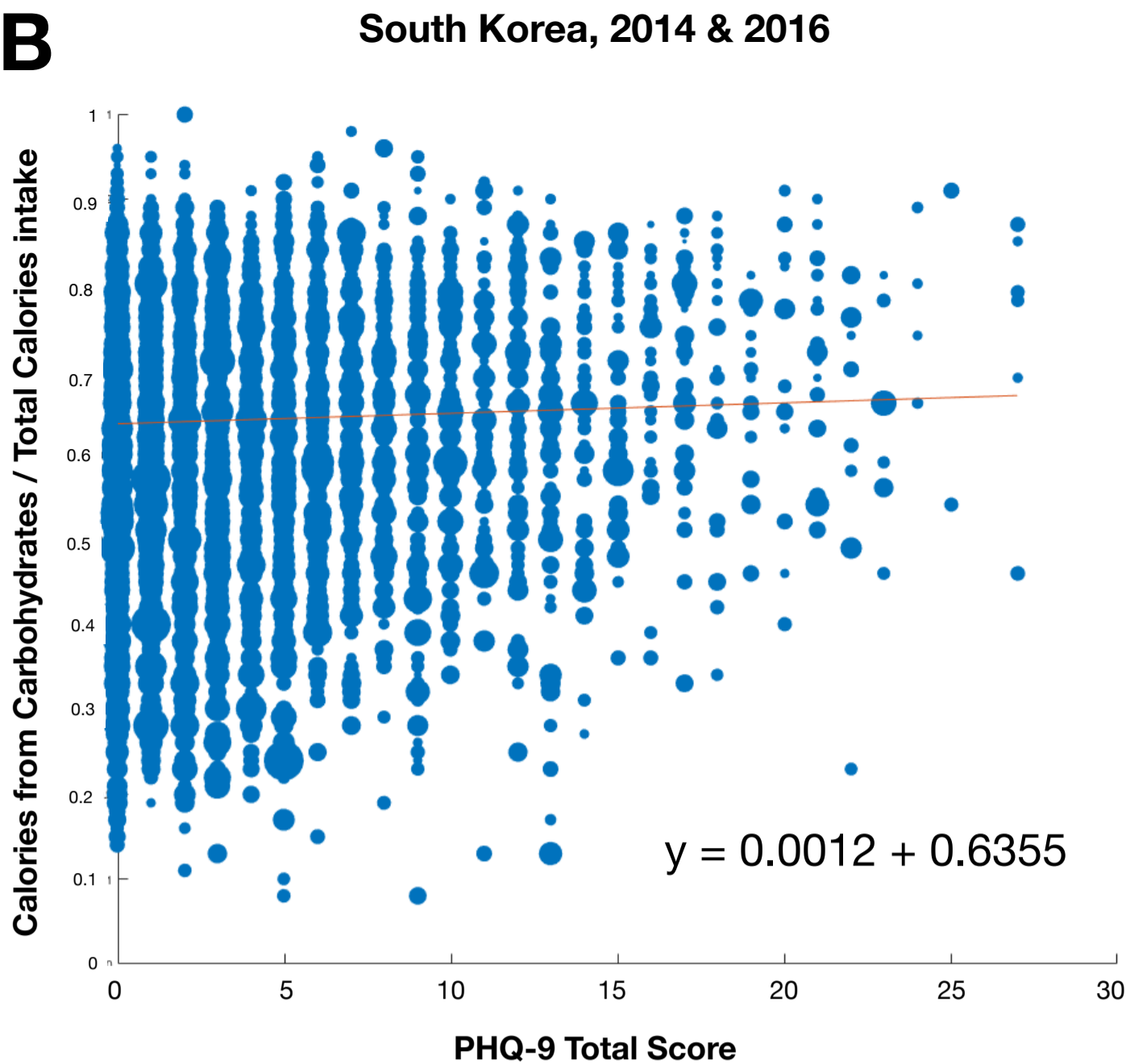

Proteins

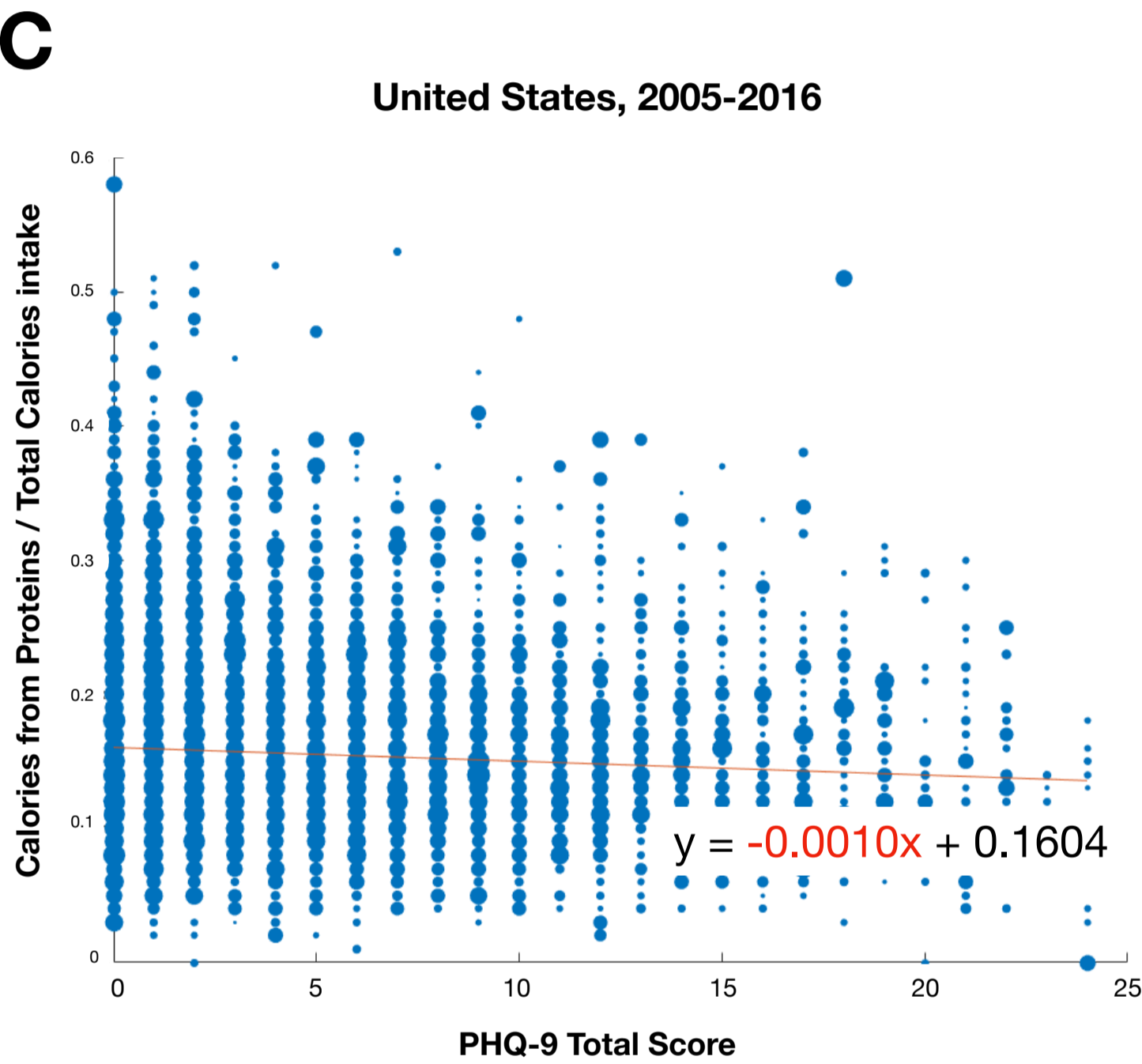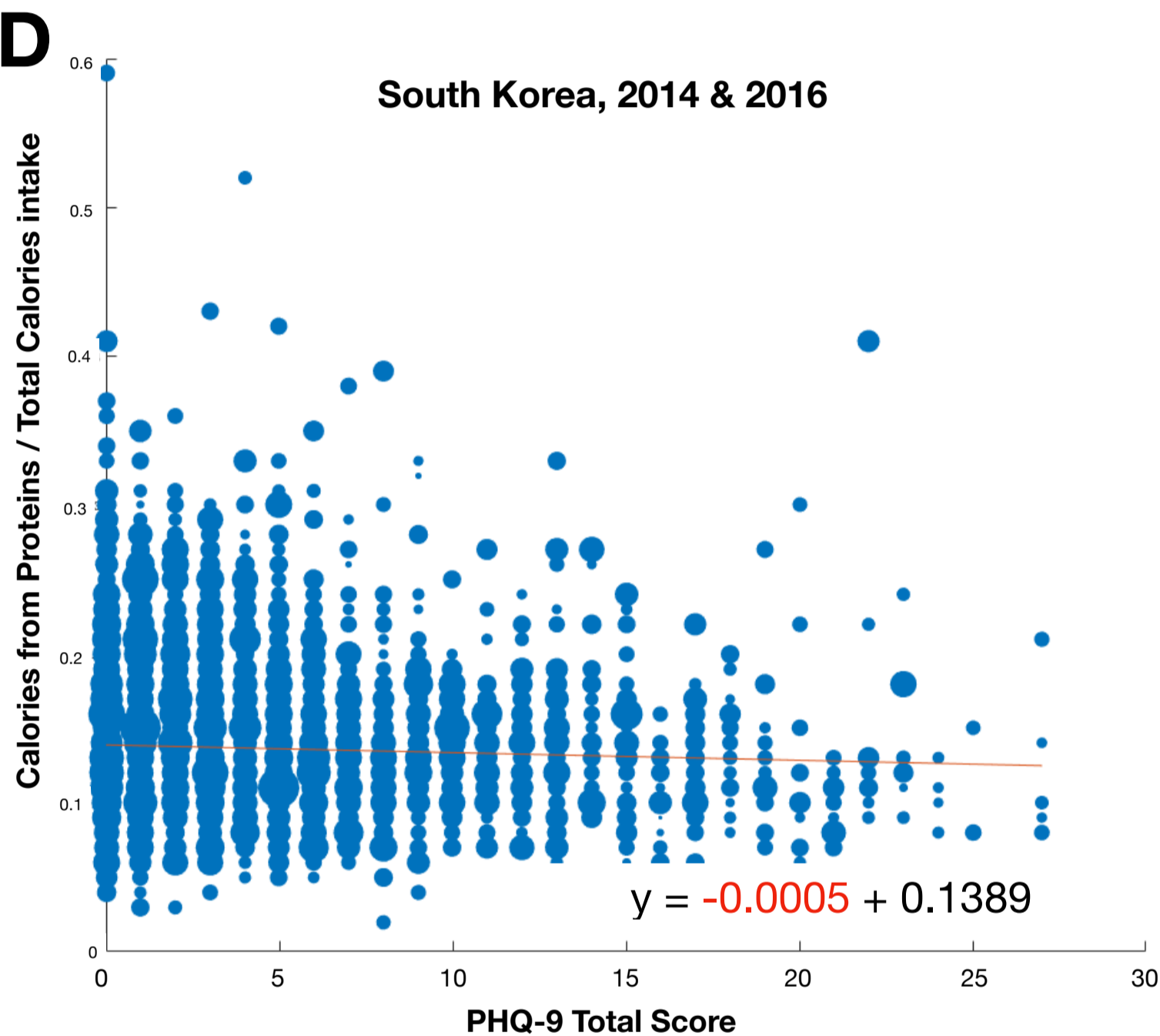

Fats

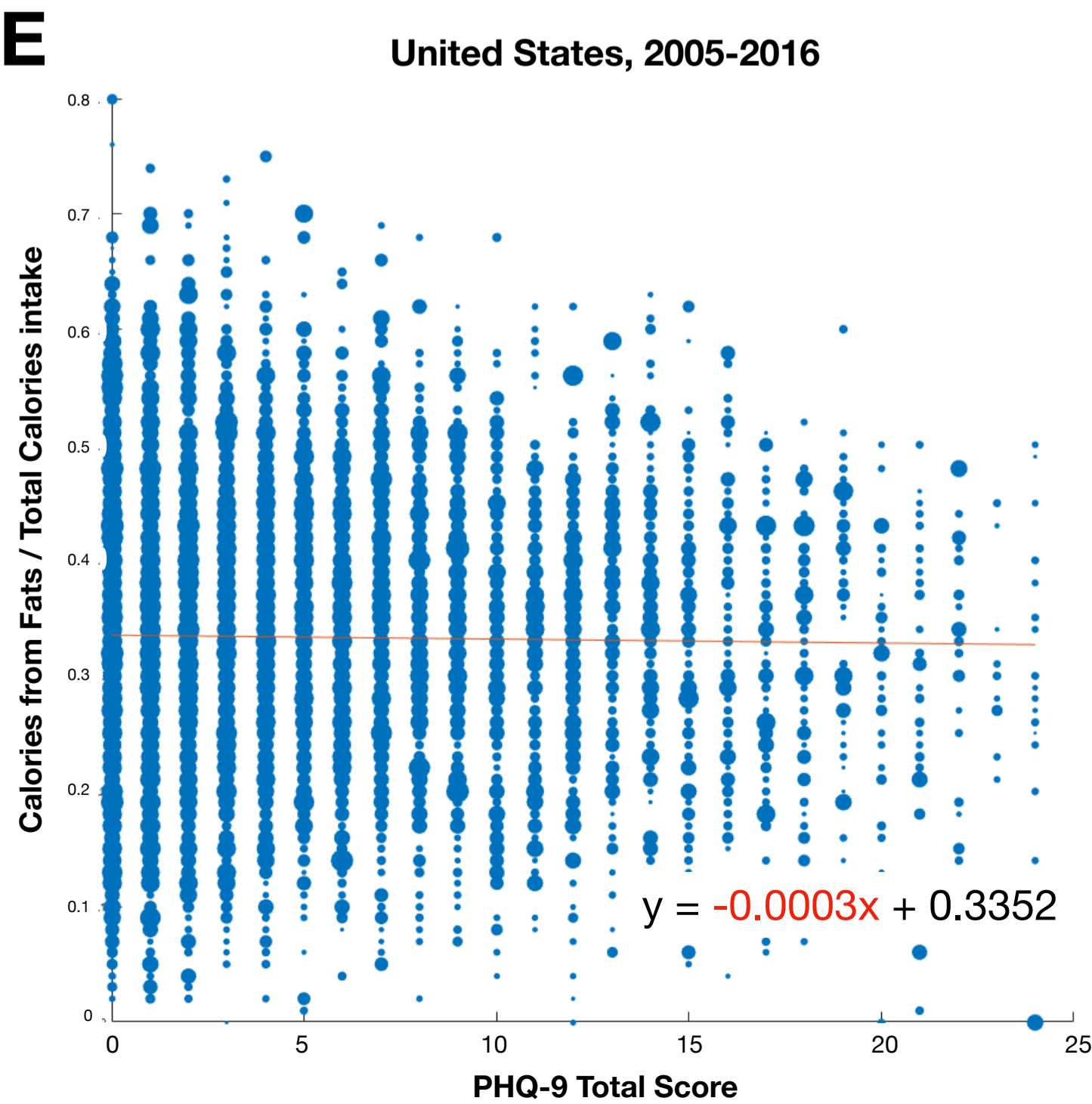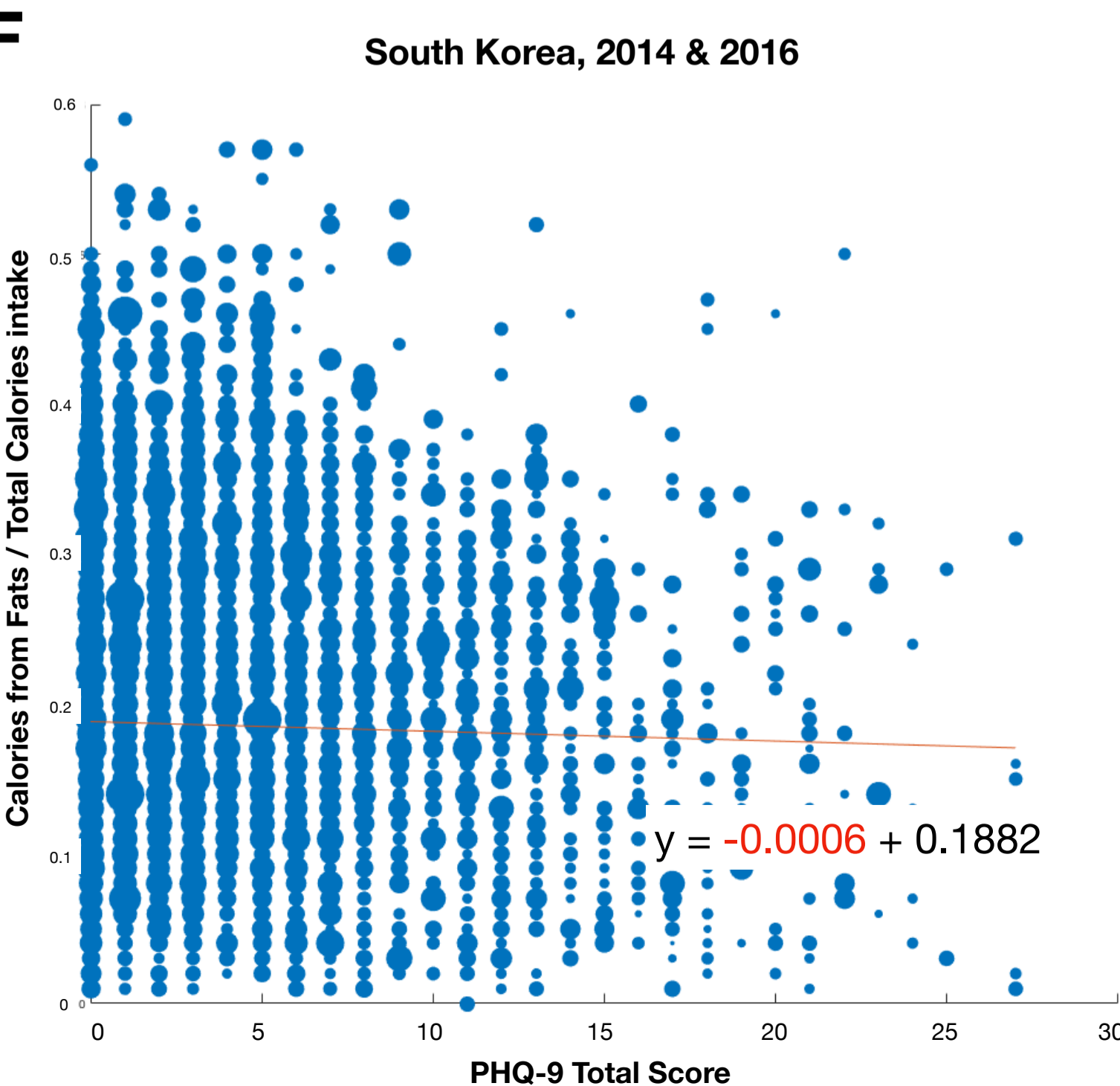

Supplement: Supplementary Figure 3 — Weighted scatter plots of PHQ-9 total score and each macronutrients intake ratio. [file Image_3.pdf]
