# Supplementary material for: Association Between Macronutrients Intake and Depression in the United States and South Korea
Source: Front Psychiatry. 2020 Mar 17;11:207. doi: 10.3389/fpsyt.2020.00207 (PMC7090018; doi:10.3389/fpsyt.2020.00207)
Supplement: Supplementary file 4 [file Table_1.docx]

**STable 1. Adjusted^a^ association between Each Nutrient and Depression in the United States and South Korea**

|  | | **Carbohydrate^b^** | | | **Protein^b^** | | | **Fat^b^** |
| --- | --- | --- | --- | --- | --- | --- | --- | --- |
| **Nutrient Intake Status** | |  |  |  |  |  |  |  |
| **United States** | | |  | | |  |  | |
|  | Normal Intake of Carbohydrate^c^ | | – | **0.603 (0.498-0.729)^e^** | | | 1.074 (0.919-1.255) | |
| **South Korea** | | |  |  | | |  | |
|  | Normal Intake of Carbohydrate^d^ | | – | 0.569 (0.310-1.042)^f^ | | | 1.346 (0.998-1.816) | |

^a^ Adjusted for age, gender, income, BMI, hypertension, dyslipidemia, diabetes and chest pain.

^b^ Odds ratio for 10% increase of each nutrient and depression.

^c^ AMDR (Acceptable Macronutrient Distribution Range) for carbohydrate: 45 to 65% of total calories intake per day.

^d^ KDRIs (Dietary Reference Intakes for Koreans) for carbohydrate: 55 to 70% of total calories intake per day.

^e^ p < 0.001

^f^ p = 0.067
